# Composite Resins Impregnated by Phosphorus Organic Extractants for Separation of Rare Earth Elements from Nitrate-Based Leachate of Permanent Magnets

**DOI:** 10.3390/ma16196614

**Published:** 2023-10-09

**Authors:** Olga V. Kovalenko, Vladimir E. Baulin, Yuri M. Shulga, Dmitriy V. Baulin, Gennady L. Gutsev, Aslan Yu. Tsivadze

**Affiliations:** 1 A. N. Frumkin Institute of Physical Chemistry and Electrochemistry, Russian Academy of Sciences, Leninsky Prospect 31, Building 4, 119071 Moscow, Russia; olga_smit@mail.ru (O.V.K.); mager1988@gmail.com (V.E.B.); badmitriy@gmail.com (D.V.B.); atsiv43@mail.ru (A.Y.T.); 2 Federal Research Center for Problems of Chemical Physics and Medicinal Chemistry, Russian Academy of Sciences, Ac. Semenov Avenue 1, 142432 Moscow, Russia; yshulga@gmail.com; 3 Department of Physics, Florida A&M University, Tallahassee, FL 32307, USA

**Keywords:** extraction chromatography, solvent impregnated resins, IR spectra, organophosphorus extractants, permanent magnets, Pr(III), Nd(III), Dy(III)

## Abstract

Composite resins impregnated by different organophosphorus extractants were developed and used for the extraction chromatography recovery of rare earth elements from nitrate-based leachate of NdFeB permanent magnets. The influence of different factors on recovery of Nd(III) and Fe(III), as the most difficult to separate elements, by developed resins was studied. The influence of extractant structure, the composition of feed solutions, and concentrations of HNO_3_ and NH_4_NO_3_ on the recovery of Fe(III) and Nd(III) by prepared resins were considered. The best recovery of Nd(III) was shown by resin impregnated with N,N-dioctyl (diphenylphosphoryl) acetamide. For this material, sorption characteristics (values of the distribution coefficient, capacity, and the Nd(III)/Fe(III) separation factor) were obtained, and the reproducibility of the loading–stripping process was evaluated. This resin and its precursors were characterized by IR spectroscopy. It was found that the developed resin is more efficient for Nd(III) recovery than resin impregnated with TODGA. An effective approach to the Nd(III)/Fe(III) separation with developed resin in nitrate solution was proposed. This approach was used for recovery of Pr(III), Nd(III), and Dy(III) from the nitrate-based leachate of NdFeB magnets by the developed resin. The final product contained 99.6% of rare earths.

## 1. Introduction

Due to their unique physical properties, rare earth elements (REEs) are widely used in various fields of industry, such as metallurgy; electronics; petrochemistry; nuclear power; medicine; and the production of magnetic, optical, laser, and fluorescent materials [1]. The ever-increasing demand for REEs has led to the depletion of natural reserves and the necessity of obtaining them from secondary sources, such as permanent magnets [2], waste fluorescent lamps [3], coal combustion products [4], industrial dumps [5], etc. However, REE recovery from these sources is a difficult task because of multicomponent compositions and the extremely low content of rare earth metals in these sources.

Among industrially consumed products containing rare earth elements, permanent magnets have the greatest economic prospects, and NdFeB magnets are the strongest of all known permanent magnets. They are widely used in consumer electronics, screw turbines, electric motors, production of radio equipment, measuring instruments, and medical equipment [6]. These magnets are important industrially consumed products of REE such as Pr(III), Nd(III), and Dy(III) [7]. One can expect that the intensive development of industry and instrumentation will lead to ever-growing demand for NdFeB magnets, and this should rise to 240–633 kt by 2030 [8]. Obviously, the value of NdFeB magnets as industrially consumed products of REEs will also increase.

Currently, liquid–liquid extraction methods have received widespread practical application for REE recovery from acid leachates of permanent magnets. The advantages of these methods are the synthetic availability of organic extractants, the high extraction degree of REEs, and the ability to extract individual REEs. Based on mechanisms of metal extraction, organic extractants can be divided into the following groups:

1. Acid extractants such as di(2-ethylhexyl)phosphoric acid (HDEHP) [9,10], 2-ethylhexylphosphonic acid mono-2-ethylhexyl ester (EHEHPA, PC88A) [11,12], bis(2,4,4-trimethylpentyl)phosphinic acid (Cyanex 272) [13], and dioctyldiglicol amic acid (DODGAA) [14];
2. Neutral extractants such as tributhyl phosphate (TBP) [15,16], phosphine oxides of different structure (TOPO, Cyanex 923) [17,18], and N′,N′,N,N–tetraoctyldiglycol amide (TODGA) [19,20];
3. Amines and salts of quaternary ammonium bases [21,22].

However, extraction methods also have significant disadvantages such as low selectivity, the existence of multiple stages in the separation process, third-phase or crud formation, the toxicity and flammability of extractants, the large consumption of organic solvents, the difficulty of the separation of organic and aqueous phases, the complicated regeneration of extractants, and environmental pollution with large amounts of liquid organic wastes. An alternative approach that lacks these disadvantages is extraction chromatography, which uses solvent impregnated resins (SIRs), where extractant is retained in pores and on the surface of inert carriers due to non-covalent forces. The efficiency of the extraction chromatography method for REE recovery from solutions of various compositions has been demonstrated in numerous studies [23,24,25,26,27,28]. To obtain impregnated resins, it is important to search for new extractants, which could increase the capacity of resins toward REE extraction and improve their separation efficiency with respect to other elements that are included in compositions of NdFeB magnets. When extracting REEs from acid leachates of NdFeB magnets, the most difficult point is to separate Fe(III) and Nd(III). Therefore, the main task in the development of new resins is to explore the recovery and efficiency of the separation of these metals.

The search for extractants suitable for preparing SIRs was started with the analysis of literature data on the liquid–liquid extraction of REEs via these compounds. Numerous studies have shown the efficiency of diphosphine dioxides for the extraction of REEs from solutions of various compositions. Turanov A.N. with collaborators have studied patterns of REE extraction via solutions of tetrasubstituted methylenediphosphine dioxides, including tetraphenylmethylenediphosphine dioxide (compound **I**), in 1,2-dichloroethane from nitric acid media [29]. It was shown that these compounds effectively extract REEs in the range of HNO_3_ concentration from 0.5 to 3.0 mol/L, and values of REE distribution coefficients decrease with an increase in atomic number. It was found that the replacement of alkyl substituents in diphosphine dioxide molecules with aryl ones leads to an increase in the efficiency of REE extraction, which is probably due to the manifestation of the aryl strengthening effect [30]. These compounds extract REEs according to the solvate mechanism which is accompanied with the formation of di- or tri-solvates, as shown by the following reaction:

Ln^3+^_aq_ + 3NO_3_^−^_org_ + xL_org_ ⇔ Ln(NO_3_)_3_L_xorg_ (1)

where the symbols “aq” and “org” denote aqueous and organic phases, respectively, x = 2 or 3.

**Figure 1:**
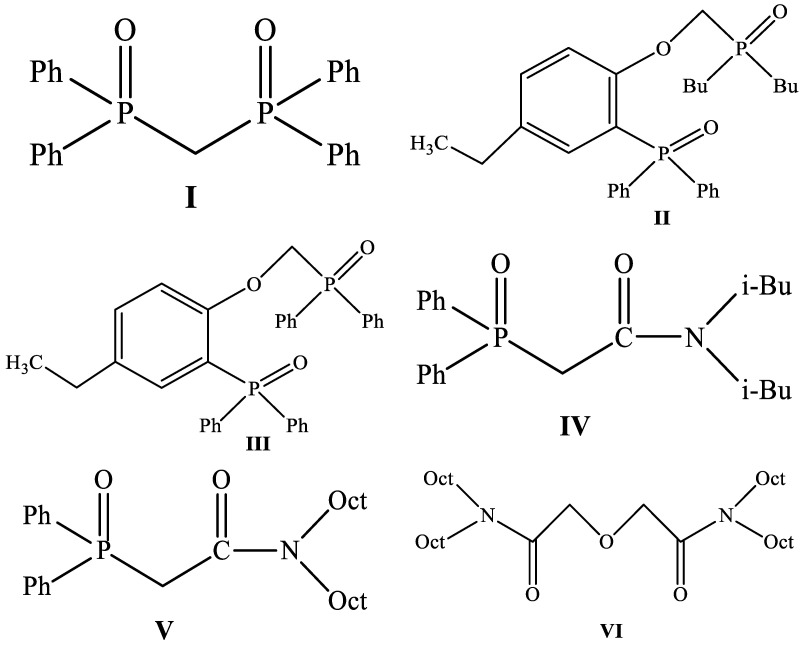
The structures of extractants used in this research.

Additionally, it was found that compounds **I** and **III** (Figure 1) are more efficient than TODGA in terms of the extraction efficiency of REEs from nitric acid media [31]. Formerly, compound **I** was used as a component of impregnated resin for REE extraction from solutions obtained after the dissolution of low-alloyed steels. The high efficiency of this resin in the separation of REEs from a number of transition metals including Fe(III) was demonstrated in [32].

Compounds **II** and **III** (Figure 1) proved to be effective extractants for the separation of REEs, U(VI), and Th(IV) in nitric acid solutions. It was established that the replacement of OCH_2_P(O)Bu_2_ by OCH_2_P(O)Ph_2_ in molecules of these extractants leads to a rise in values of REE distribution coefficients, whereas the values of separation factors for U(VI)/REE and Th(IV)/REE change in the opposite way, which apparently is due to the aryl strengthening effect. These compounds extract REEs according to the solvate mechanism with mono- or dissolvate formation [33]. The recovery of Nd(III) with resins impregnated by a mixture of compound **III** and ionic liquid [C_4_mim]^+^[Tf_2_N]^−^ in nitric acid solutions was also studied. It was shown that the introduction of ionic liquid in resin composition leads to significant enhancement in values of Nd(III) distribution coefficients compared to resins containing only compound **III**. The highest value of the Nd(III) distribution coefficient was obtained for resins containing 40 wt% of a mixture of compound **III** and [C_4_mim]^+^[Tf_2_N]^−^ in the ratio of 2:1. Moreover, this resin was more efficient than the resin impregnated by the mixture of Cyanex 923 and [C_4_mim]^+^[Tf_2_N]^−^ in terms of the efficiency of Nd(III) recovery [25]. However, the ability to separate Nd(III) from Fe(III) using this resin has not been studied.

The influence of the structure of diphenyl(dialkylcarbamoylmethyl)phosphine oxides (CMPOs) (compounds **IV** and **V**) (Figure 1) on the efficiency of REE recovery from nitric acid media was examined in [34]. It was shown that resins impregnated by CMPOs effectively extract REEs from HNO_3_ with concentrations from 1.0 to 7.0 mol/L mainly in the form of dissolvates according to relation (1), where x = 2 and L is CMPO.

Some CMPOs can extract REEs in the form of trisolvates. It was found that an increase in the length of the hydrocarbon chain from two to six in CMPO molecules leads to a rise in values of REE distribution coefficients. During the transition from a hexyl substituent to an octyl one, the recovery of REEs slightly decreases, which is likely due to the influence of steric factors. For all studied CMPOs, the REE recovery efficiency declines insignificantly during the transition from La(III) to Lu(III). Despite the fact that patterns of REE recovery via resins based on CMPOs have been studied in detail, there is practically no information on the efficiency of separation between REEs and Fe(III), as well as other transition metals via these resins. Therefore, bidentate organophosphorus extractants are promising components of impregnated resins for the recovery of REEs from solutions of different compositions.

The present research is aimed at the search for effective extractants and the development of new resins for the recovery of rare earth elements from nitrate-based leachates of NdFeB permanent magnets. The choice of effective resins is based on the assessment of separation efficiency for Fe(III) and Nd(III), since they are the elements whose separation is the most difficult to perform. The main characteristics such as distribution coefficients, capacity, separation factors, and reproducibility for the developed resin were determined, and its efficiency in REE recovery from leachates of NdFeB magnets was evaluated.

## 2. Experimental Section

### 2.1. Solutions and Reagents

Feed solutions of Nd(III) and Fe(III) were prepared by dissolving precisely weighed portions of Nd(NO_3_)_3_·6H_2_O and Fe(NO_3_)_3_·9H_2_O (Aldrich, MD, USA) purity >99.9%) in HNO_3_ solutions of different concentrations. Nitric acid solutions were prepared by diluting concentrated HNO_3_ with distilled water. The HNO_3_ concentrations in prepared solutions were determined via titration with a standard solution of NaOH in the presence of bromothymol blue. The concentrations of Nd(III) and Fe(III) in eluates were determined spectrophotometrically using Arsenazo M [35] and 10%-KSCN [36]. All used reagents were analytical grade. Nitrate-based leachate was prepared by dissolving a NdFeB magnet (Table 1) in HNO_3_ diluted 1:1.

**Table 1:** Composition of spent magnet [37].

Element	Content (wt%)
Fe	61.1 ± 1.0
Nd	25.4 ± 0.6
B	1.00 ± 0.02
Al	0.95 ± 0.16
Co	1.42 ± 0.07
Dy	1.08 ± 0.27
Pr	2.62 ± 0.17
Mn	0.15 ± 0.01
Cu	0.22 ± 0.05
Ni	2.03 ± 0.23

The leachate was neutralized with a solution of NH_4_OH to pH of ~6.5–7.0. The composition of the obtained solution is presented in Table 2.

**Table 2:** Composition of neutralized nitrate-based leachate.

Element	Concentration, mg/L
Fe	575 ± 1
Nd	259.0 ± 0.9
B	11.2 ± 0.1
Co	48.8 ± 0.9
Pr	34.1 ± 0.9
Dy	4.05 ± 0.05
Al	5.60 ± 0.07
Mn	0.81 ± 0.03
Cu	0.60 ± 0.02
Ni	0.39 ± 0.03

The values of metal concentration in the nitrate-based leachate were determined with the help of Thermo Elemental—X7 Quadrupole ICP-MS (Thermo Fisher Scientific, Waltham, MA, USA).

### 2.2. Synthesis

A number of organophosphorus compounds **I**–**V** (Figure 1) were synthesized for the preparation of impregnated resins. Tetraphenylmethylenediphosphine dioxide (I) was synthesized using the method in [32], dibuthyl((2-(diphenylphosphoryl)-4-ethylphenoxy(methyl)phosphine oxide (II) was prepared using the method described in [33], and ((2-diphenylphosphoryl)-4-ethylphenoxy(methyl)diphenylphosphine oxide (III) was synthesized using the method in [25]. The synthesis methods for N,N,-diisobuthyl(diphenylphosphoryl)acetamide (IV) and N,N-dioctyl(diphenylphosphoryl)acetamide (V) are described in [38].

During the synthesis of compounds **I**–**V**, the compositions of reaction mixtures were controlled using thin-layer chromatography on Silufol plates (Merck, Rahway, NJ, USA). The mixture of CHCl_3_ and i-C_3_H_7_OH in a ratio of 10:1 was used as an eluent. The manifestation of chromatograms was carried out in iodine vapors. N′N′,N,N–tetraoctyldiglycol amide (VI) is provided by Sorbent Technology company (Moscow, Russia).

The structure and purity degree of synthesized compounds were determined by using NMR spectroscopy and the elemental analysis data. The content of C and H was determined by standard methods using a Carlo Erba CHN analyzer (Erba Group, Brno, Czech Republic). NMR spectra were recorded using a CXP-200 or Bruker-DXP-200 (200 MHz) instrument (Bruker, MA, USA) with tetramethylsilane as the internal standard, while 85% H_3_PO_4_ was used as the reference in the ^31^P NMR spectra.

### 2.3. Extraction of REEs

The extraction of Pr(III), Nd(III), and Dy(III) in a solution of NH_4_NO_3_ with a concentration of 1.0 mol/L was carried out by using solutions of compound **V** in n-nonane. Extractant solutions were prepared by dissolving precisely weighed portions of compound **V** in n-nonane. Feed solutions of Pr(III), Nd(III), and Dy(III) in a solution of NH_4_NO_3_ were prepared by dissolving precisely weighed portions of Pr(NO_3_)_3_·6H_2_O, Nd(NO_3_)_3_·6H_2_O, Dy(NO_3_)_3_·5H_2_O, and NH_4_NO_3_ (Aldrich, purity >99.9%) in distilled water. The initial concentration of rare earth elements was 1 × 10^−6^ mol/L. Phases were brought into contact with each other in a rotor-stirring apparatus at room temperature at a stirring speed of 60 rpm for 1 h. By special experiments, it was shown that this time is enough to reach equilibrium in the extraction of REEs. It was found that after 45 min, the concentration of REEs in the aqueous phase was not changed. We used V_org_ = V_aq._ The values of initial and equilibrium concentrations of REEs in the aqueous phase were determined using the spectrophotometric method with Arsenazo M. The values of distribution coefficients (D) of metals were calculated from the following Equation (2):

D = (C_0_ − C_eq_)/C_0_ (2)

where C_0_ and C_eq_ are initial and equilibrium concentrations of metals in the aqueous phase in mg/L, and V_org_ and V_aq_ are the volumes (in mL) of the organic and aqueous phases, respectively.

### 2.4. Preparation of Resins

The studied resins were prepared using the method described in [39] and characterized in Table 3. Weighed portions of compounds **I**–**V** (Figure 1) were dissolved in 30 mL of CHCl_3_ and mixed with a suspension of a copolymer of styrene with divinylbenzene LPS-500 (the specific surface area was 570 m^2^/g, the diameter of pores was 3–50 nm, and the size of particles was 40–70 µm; provided by RossPolimer company, Moscow, Russia) in 20 mL of CHCl_3_. The resulting mixture was stirred in the rotating flask of a rotary evaporator, and then, CHCl3 was removed in vacuum at 50 °C. The resulting residue was kept at 40–50 °C for 30 min at 1 mm Hg to completely remove CHCl_3_. The SIR containing TODGA was prepared in a similar way.

### 2.5. IR Spectra

The IR spectra obtained with a resolution of 1 cm^−1^ and 32 scans were recorded at room temperature in the range of 450–4000 cm^−1^ on a Perkin-Elmer “Spectrum Two” FT-IR spectrometer (Waltham, MA, USA) with an ATR attachment.

### 2.6. Chromatographic Equipment

The recovery of Nd(III) and Fe(III) was studied in dynamic mode using a chromatographic device manufactured by Knauer which consists of three high-pressure pumps, a dosing tap, chromatography column, and spectrophotometric detector. The recovery of metals was carried out using a plastic column with a length of 100 mm and a 4 mm internal diameter. The column was packed with resins using the “dry method”, i.e., by loading dry resin inside the column in small portions and pressing them down with a glass rod. The physical constants of the prepared columns are shown in Table 4.

### 2.7. Dynamic Uptake of Nd(III) and Fe(III)

**Figure 2:**
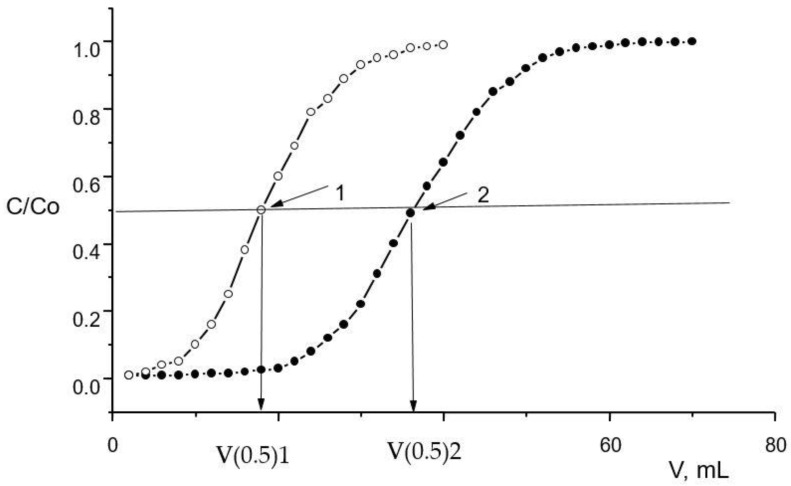
The calculated values of metal distribution coefficients using frontal loading curves; C/C_0_ is the ratio of metal concentrations in eluate and feed solutions [40]. 1 and 2 are frontal curves for two different metals.

The chromatographic column packed with 600.8 ± 0.7 mg of SIRs (Table 3) was washed with a peristaltic pump with a HNO_3_ solution of a chosen concentration with a flow rate of 1.0 mL/min for 1.0 h. Then, Nd(III) or Fe(III) solutions with concentrations of 580.5 ± 0.5 mg/L and 260.5 ± 0.3 mg/L, respectively, were constantly passed through the column until SIR saturation was completed with a flow rate of 1.0 mL/min. Concentrations of Nd(III) and Fe(III) in eluates which left the column were automatically determined using the spectrophotometric method. The frontal loading curves shown in Figure 2 were used to calculate the values of distribution coefficients and the capacity of SIRs. Experiments were performed at room temperature.

The dynamic distribution coefficients (Kd) were calculated from Equation (3) [40]:

Kd = V_0.5_/m_e_ (3)

where V_0.5_ is the volume of solution until the half breakthrough of metal, mL; m_e_ is the mass of extractant in resin, g. The values of separation factors (β) were calculated from the following Equation (4):

β = Kd_2_/Kd_1_ (4)

where Kd_1_ and Kd_2_ are distribution coefficients of separating metals.

### 2.8. Separation of Fe(III) and Nd(III)

The column packed with 600 ± 3 mg of resin SIR 5 was washed with a solution of NH_4_NO_3_ with a concentration of 1.0 mol/L for 1.0 h. Then, a solution containing both Fe(III) and Nd(III) in NH_4_NO_3_ with a concentration of 1.0 mol/L was passed through the column to the full saturation of resin with metals. Fe(III) and Nd(III) concentrations in the feed solution were 580.5 ± 0.5 mg/L and 260.5 ± 0.3 mg/L, respectively. Further, the resin was consistently washed with a solution of NH_4_NO_3_ with a concentration of 1.0 mol/L for 10 min and distilled water for 30 min. The flow rate of all solutions was 1.0 mL/min. Concentrations of Fe(III) and Nd(III) were determined using the spectrophotometric method. All experiments on the recovery and separation of Nd(III) and Fe(III) were carried out at room temperature.

### 2.9. Recovery of Rare Earth Elements from Nitrate-Based Leachate of NdFeB Permanent Magnet

The recovery of rare earth elements from nitrate-based leachate of a NdFeB permanent magnet using resin SIR 5 was carried out using the method described in Section 2.8. Metal concentrations in nitrate-based leachate were determined using the method of ICP-MS (see above).

## 3. Results and Discussion

### 3.1. The Choice of Extractant for Preparation of Resins

**Figure 3:**
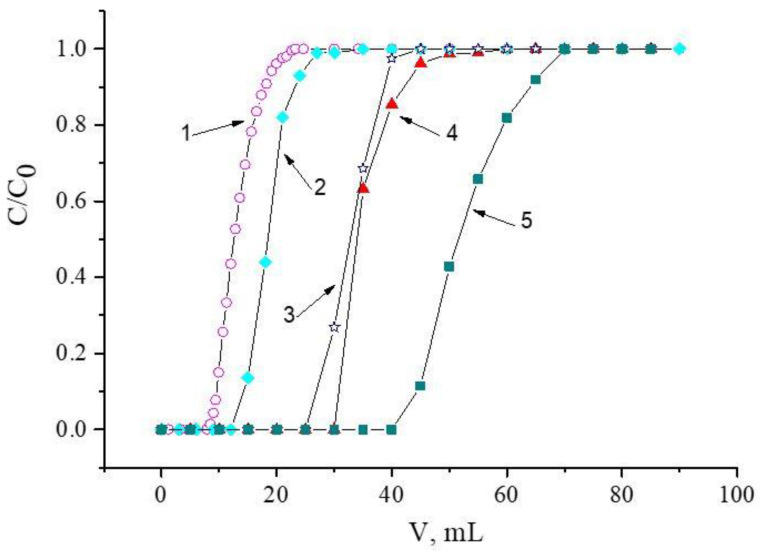
Frontal loading curves of Nd(III), obtained with the following resins: 1 is for SIR 1; 2 is for SIR 2; 3 is for SIR 3; 4 is for SIR 4; 5 is for SIR 5. The SIR content is 600 ± 3 mg; the concentration of Nd(III) in the feed solution is 260.5 ± 0.3 mg/L; the flow rate is 1.0 mL/L.

Since acidic solutions are obtained during the processing of NdFeB magnets, the recovery of Nd(III) and Fe(III), which are the most difficult to separate, on developed resins from nitric acid solutions was explored first. The choice of the most suitable resin was based on the results obtained when using HNO_3_ with a concentration of 1.0 mol/L (Figure 3).

One can see from Figure 3 that the best recovery of Nd(III) is achieved by using SIR 5. Resins containing carbamoylmethylphosphine oxides (SIR 4 and SIR 5) retrieve Nd(III) more efficiently than those based on diphosphine dioxides (SIR 1, SIR 2, and SIR 3). It is worth noting that the replacement of alkyl substituents in compound **II** with phenyl ones in compound **III** led to an increase in the value of the Nd(III) distribution coefficient, which is most likely due to the aryl strengthening effect. Earlier, such an effect was observed during the extraction of REEs from nitric acid media by using neutral organophosphorus compounds [41]. Additionally, an increase in the length of carbon substituents at the N atom led to the significant enhancement of the Nd(III) Kd value, which was probably due to the greater lipophilicity of compound **V** compared to that of organic ligand **IV**. The values of the distribution coefficients and capacities of the studied resins via Nd(III) are presented in Table 5. Note that Fe(III) was not extracted using any of these resins under these conditions. Based on the obtained results, SIR 5 was selected for further experiments.

### 3.2. IR Spectra

**Figure 4:**
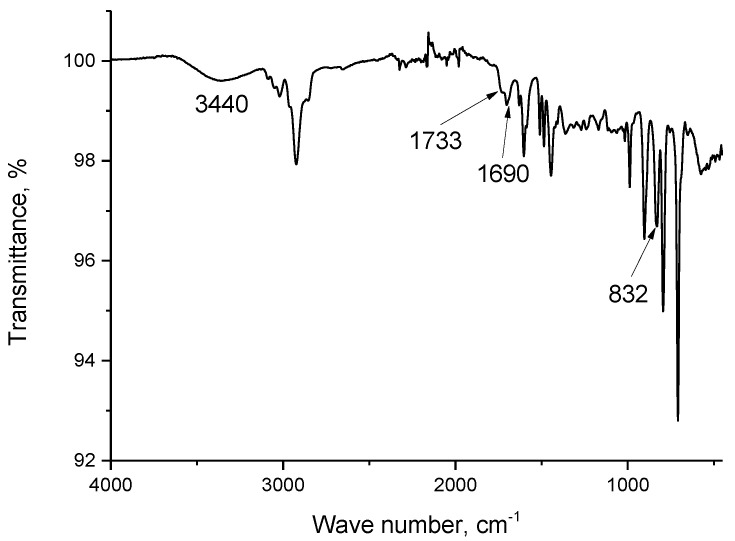
The IR-spectrum of LPS-500.

**Figure 5:**
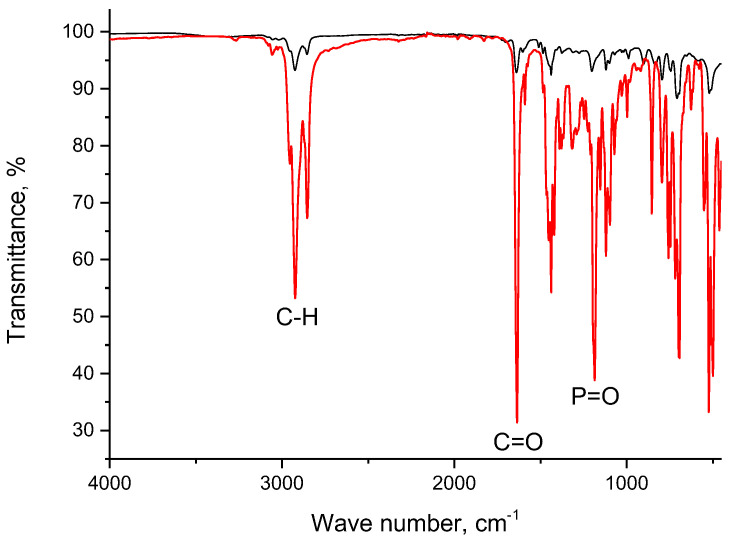
IR spectra of compound **V** (red line) and SIR 5 (black line).

**Figure 6:**
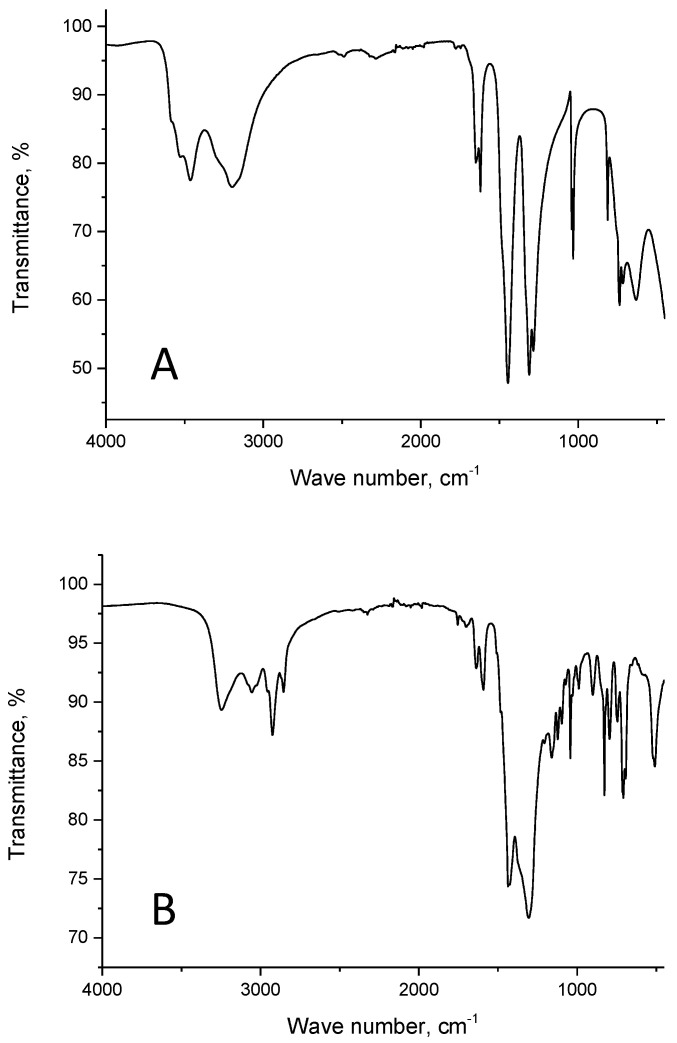
IR spectra of Nd(NO_3_)_3_∙6H_2_O (**A**) and SIR 5 saturated with Nd(III) (**B**).

**Figure 7:**
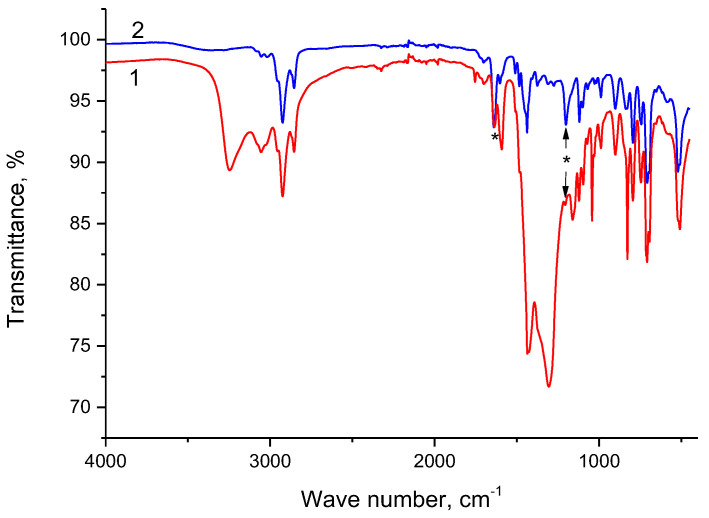
IR spectra of SIR 5 saturated with Nd(III) (1), and initial SIR 5 (2). * mean peaks corresponding to P=O and C=O groups.

For a better understanding of Nd(III) recovery by resin SIR 5, IR spectra of LPS–500, compound **V**, Nd(NO_3_)_3_∙6H_2_O, resin SIR 5, and resin SIR 5 saturated with Nd(III) were recorded as shown in Figure 4, Figure 5, Figure 6 and Figure 7. The properties of copolymers of styrene with divinylbenzene and their IR spectra were studied in a number of publications [42,43,44,45]. The IR spectrum of LPS-500 copolymer used as inert carrier in the developed resins is shown in Figure 4.

The spectrum has a wide absorption band with maximum at 3440 cm^−1^, which can tentatively be attributed to the O–H bond vibrations. However, there are no OH groups in the polymer structure, and one can attribute this peak either to adsorbed water molecules or to products of polymer partial oxidation. The partial oxidation of LPS-500 can also be confirmed by the presence of bands at 1733 cm^−1^ and 1690 cm^−1^, which can be attributed to carboxyl and carbonyl groups. Absorption bands in the range of 1100–1300 cm^−1^ are due to vibrations of groups –C–OH and –C–O–C [43]. A wide feature at 832 cm^−1^ means that LPS-500 is amorphous [44].

The spectra of compound **V** and SIR 5 are presented in Figure 5. In the spectrum of compound **V**, there are peaks corresponding to vibrational frequencies of C=O and P=O bonds at 1636 cm^−1^ and 1186 cm^−1^, respectively. Additionally, the intensities of peaks corresponding to vibrational frequencies of aliphatic C–H bonds (peaks at 2954 cm^−1^, 2924 cm^−1^, and 2854 cm^−1^) significantly exceed the intensities of peaks above 3000 cm^−1^ corresponding to aromatic C–H bonds. In the case of SIR 5, this difference is slightly reduced due to contributions of polymer (the inert carrier). In general, the peak intensities of compound **V** in the resin spectrum exceed those of LPS-500, which is indicative of a high concentration of extractant in resin. It is interesting that the introduction of an extractant in the polymer carrier is accompanied by a shift in the frequency of vibrational frequencies of the C=O bond towards higher wave numbers by only 4 cm^−1^, whereas in case of the P=O bond, this shift is 16 cm^−1^.

It is known that the NO^3−^ anion has trigonal planar configuration and manifests itself in the IR spectrum as an intense peak at 1380 cm^−1^ [46]. However, in the case of a strong Metal–O bond, the anion symmetry reduces to C_2v_, and the peak corresponding to N–O vibrations becomes split. The anion can have both bidentate and bridging bonds. As a result, there is a double N=O bond, which manifests itself as a peak at about 1600 cm^−1^ in IR spectra [47]. The IR spectrum of Nd(NO_3_)_3_∙6H_2_O obtained in this research (Figure 6) matches the spectrum in [48]. In this compound, the anion is monodentate bonded to the metal [47], and the absorption band ν^as^(N–O) at 1447 cm^−1^ corresponds to the anion in the IR spectrum. It is worth noting that some peaks corresponding to vibrational frequencies of O–H bonds appear at large frequencies (3463 cm^−1^ and higher), which indicates an isolated character of these bonds.

The structure of an adsorbed neodymium complex differs from its structure in the initial state. This difference is primarily due to the absence of isolated O–H bonds with high vibration frequencies (3463 cm^−1^ and higher, Figure 6) in the structure of the adsorbed complex. In resin SIR 5, saturated with neodymium, all O–H groups (including water molecules) participate in hydrogen bonds. Further, absorption band ν^as^(N–O) broadens and shifts towards low frequencies by approximately 10 cm^−1^. Most importantly, this absorption band is observed against the background of a broad peak, which is localized in a range from 1520 cm^−1^ to 1190 cm^−1^. This broad peak maxim, which appears in the IR spectrum at approximately 1380 cm^−1^, can be explained by the presence of free NO^3−^ anions in the adsorbed neodymium complex [46].

The IR spectra of the initial SIR 5 and SIR 5 saturated with Nd(III) are compared in Figure 7. As can be seen, the intensities of peaks corresponding to vibrational frequencies of C–H bonds (2925 cm^−1^ and 2854 cm^−1^) in both spectra are almost the same. Since Nd(NO_3_)_3_∙6H_2_O has no such bonds, these peaks are due to SIR 5. An intense peak with the maximum at 3247 cm^−1^ and a wide intense band in a range from 1520 cm^−1^ to 1190 cm^−1^ are due to the adsorbed compound Nd(NO_3_)_3_∙6H_2_O.

One can anticipate that functional groups P=O and C=O participate in complexation with Nd(III). It can be seen in Figure 7 that the intensities of the peaks corresponding to the P=O and C=O bonds (marked with asterisks) in spectrum 1 are much lower than these intensities in spectrum 2. This is most clearly manifested by a peak at 1202 cm^−1^ corresponding to the P=O bond. The relatively high intensity of a peak at 1637 cm^−1^ in spectrum 1 might be due to deformation vibrations of water molecules presented in the sample that can contribute to intensity. A decrease in intensities of peaks corresponding to the P=O and C=O bonds not accompanied by a decrease in intensities of peaks corresponding to C–H bonds can be attributed to the formation of new bonds. This is in agreement with the results of previous research [49] where the participation of P=O and C=O groups in complexation with Nd(III) was confirmed. The formation of new bonds is also indicated by the appearance of new peaks in spectrum 1 (Figure 7), which were not observed in the other spectra discussed above. New peaks located at 1754 cm^−1^ and 1592 cm^−1^ can be attributed to aliphatic anhydride or lactone (C=O bond) and to a NO_3_^−^ anion with bidentate or bridging bonds, respectively.

### 3.3. Influence of HNO_3_ Concentration

**Figure 8:**
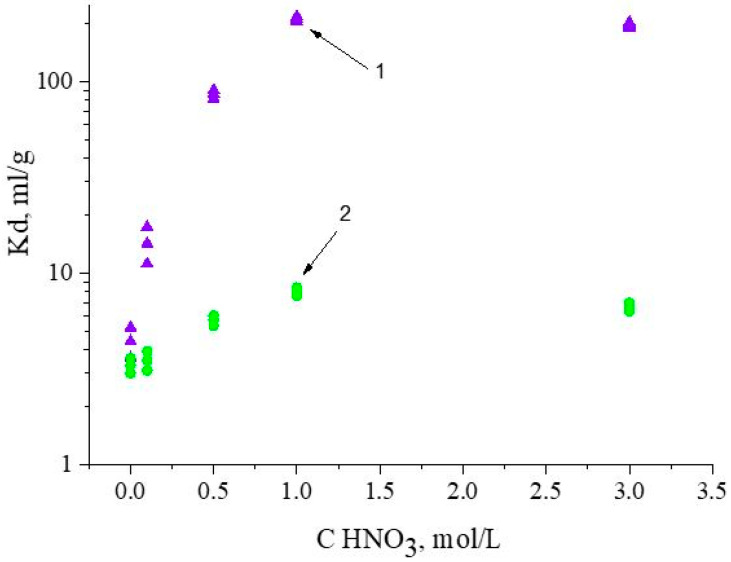
The influence of HNO_3_ concentration on values of Fe(III) and Nd(III) distribution coefficients: 1 is for Nd(III) and 2 is for Fe(III) for SIR 5. The SIR content is 600 ± 3 mg; the concentration of Nd(III) in feed solution is 260.5 ± 0.3 mg/L; the flow rate is 1.0 mL/L.

To define optimal separation conditions, the influence of the HNO_3_ concentration on the recovery of Nd(III) and Fe(III) via SIR 5 was studied, and the results are presented in Figure 8. The values of the Nd(III) distribution coefficients increase at all selected HNO_3_ concentrations, which is typical for organophosphorus neutral extractants [40]. At the same time, Fe(III) is not extracted under these conditions.

### 3.4. Influence of Composition of Feed Solutions

**Figure 9:**
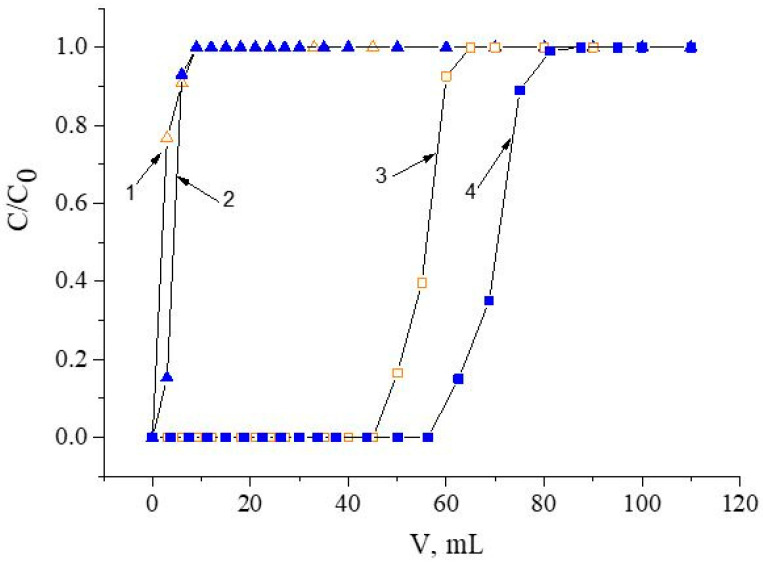
Frontal loading curves of Fe(III) and Nd(III) in solutions of HNO_3_ and NH_4_NO_3_ for SIR 5; 1 is for Fe(III) in HNO_3_; 2 is for Fe(III) in NH_4_NO_3_; 3 is for Nd(III) in HNO_3_; 4 is for Nd(III) in NH_4_NO_3_. The SIR content is 600 ± 3 mg; the concentration of Nd(III) in feed solution is 260.5 ± 0.3 mg/L; the flow rate is 1.0 mL/L.

It is known that the replacement of mineral acids with moderate concentrations (3.0–6.0 mol/L) by electrolytes of the same concentrations when metals are extracted via resins impregnated with neutral organophosphorus compounds leads to an increase in the values of both the distribution coefficients and the capacity of resins to extracted metals [40]. To enhance the efficiency of Nd(III) recovery and the separation of Nd(III) from Fe(III) by SIR 5, the electrolyte influence on metal distribution coefficients was explored. For this purpose, the recovery of Fe(III) and Nd(III) in solutions of HNO_3_ and NH_4_NO_3_ with concentrations of 1.0 mol/L is compared in Figure 9.

It was found that the replacement of HNO_3_ with NH_4_NO_3_ leads to significant enhancements in values of the Nd(III) distribution coefficient but does not affect the recovery of Fe(III). The values of the Nd(III) dynamic distribution coefficients Kd increase from 213 ± 8 to 227 ± 10 mL/g, which is probably due to the extraction of HNO_3_ via compound **V,** which leads to a decrease in the amount of free extractant in resin. The ability of neutral organophosphorus compounds to extract mineral acids has been demonstrated in [31,50]. The Kd values of Fe(III) are 8 ± 2 and 8 ± 1, respectively. Therefore, the values of the separation factors of Nd(III) and Fe(III) in HNO_3_ and NH_4_NO_3_ are 27 ± 2 and 28 ± 2, respectively. In addition, the use of NH_4_NO_3_ solutions instead of HNO_3_ solutions as eluents has different advantages: (1) the corrosion of equipment is practically eliminated; (2) methods for the determination of REE concentration in eluates and desorbates are simplified; (3) the consumption of reagents used for subsequent REE precipitation is decreased; (4) environmental damage is reduced. With the simultaneous presence of both metals in feed solution, Nd(III) adsorbs much better than Fe(III) and displaces it from sorption centers of SIR 5. Therefore, at the end of recovery, resin will be saturated mainly with Nd(III). However, a small amount of feed solution of ~1.3 mL remains in the free column volume, which can subsequently become a source of Nd(III) contamination during its further stripping.

**Figure 10:**
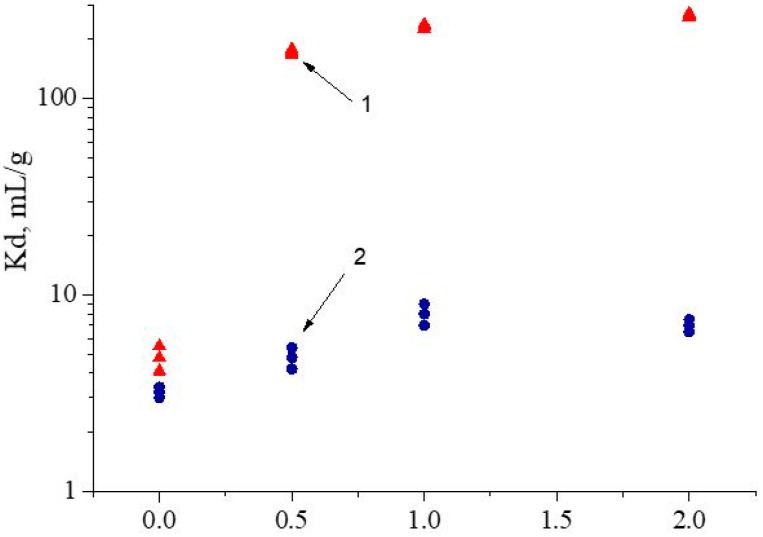
Dependence of values Kd of Fe(III) and Nd(III) on NH_4_NO_3_ concentration for SIR 5: 1 is for Nd(III) and 2 is for Fe(III). The SIR content is 600±3 mg; the concentration of Nd(III) in feed solution is 260.5 ± 0.3 mg/L; the flow rate is 1.0 mL/L.

It was shown that an increase in the concentration of NH_4_NO_3_ from 0.5 mol/L to 2.0 mol/L in feed solution leads to an increase in values of Nd(III) Kd from 173 ± 6 mL/g to 265 ± 8 mL/g, as shown in Figure 10. This is likely due to the recovery of Nd(III) via the solvation mechanism [51] according to the following reaction (5):

Nd^3+^_aq_ + 3NO_3aq_^−^ + 3L_org_ ↔ Nd(NO_3_)_3_L_3 org_ (5)

where L is compound **V**; subscripts “aq” and “org” denote aqueous and organic phases, respectively. It is worth mentioning that Fe(III) was not adsorbed under these conditions.

**Figure 11:**
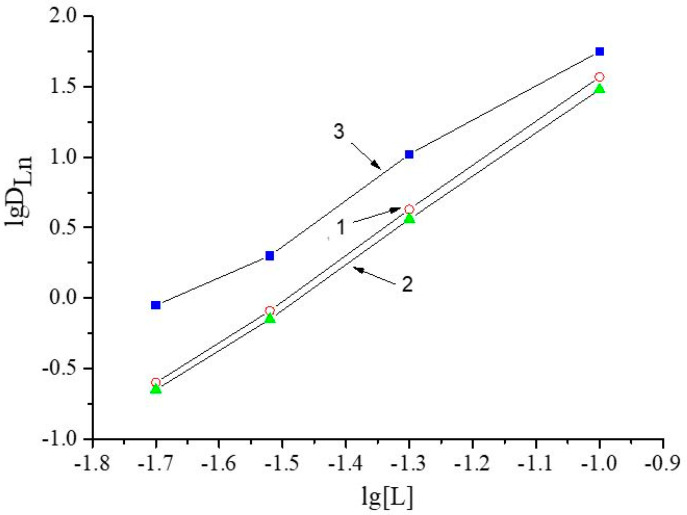
Dependence of distribution coefficient (D) on concentration of compound **V** (L): 1 is for Pr(III), 2 is for Nd(III), and 3 is for Dy(III).

The stoichiometric ratio of REEs to compound **V** in the extracted complex was determined by using the bilogarithmic equilibrium shift method during the extraction of Pr(III), Nd(III), and Dy(III) from a solution of **V** in n-nonane from NH_4_NO_3_ with a concentration of 1.0 mol/l (Figure 11).

The values of slopes for Pr(III), Nd(III), and Dy(III) are 2.8 ± 0.2, 2.9 ± 0.2, and 3.0 ± 0.3, respectively. Thus, for each of the selected REEs, the stoichiometric ratio REE: L = 1:3, which is in agreement with previously obtained data on the extraction of REEs by using solutions of carbamoylmethylphosphine oxides from nitric acid media [52].

### 3.5. Reproducibility

Our study of the recovery reproducibility of Nd(III) in NH_4_NO_3_ of 1.0 mol/L in three repeated experiments performed with the same resin has shown that values of Nd(III) distribution coefficients and the capacity of resin for Nd(III) recovery practically do not change (Table 6), which makes it possible to reuse this resin at least three times.

### 3.6. Comparison with TODGA

**Figure 12:**
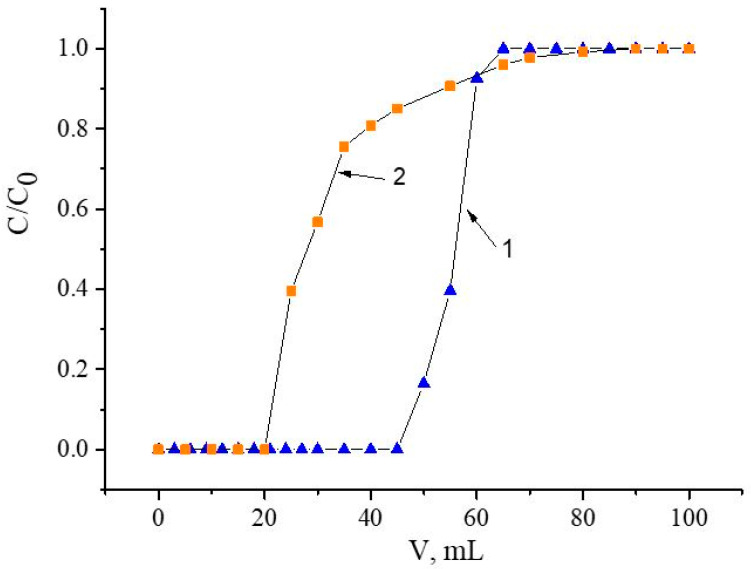
Frontal loading curves of Nd(III) in NH_4_NO_3_ of 1.0 mol/L: 1 is for SIR 5 and 2 is for SIR 6. The SIR content is 600 ± 3 mg; the concentration of Nd(III) in feed solution is 260.5 ± 0.3 mg/L; the flow rate is 1.0 mL/L.

Among various resins used to extract REEs and separate them from other related metals, resins impregnated with N′,N′,N,N–tetraoctyldiglycol amide (TODGA) were most often used [53,54,55]. The recovery of Nd(III) using resins impregnated with compound **V** (SIR 5) and TODGA (SIR 6) was studied under the same conditions (Figure 12).

It was found that the efficiency of resin SIR 5 with respect to Nd(III) recovery is superior to SIR 6 impregnated with TODGA. The sorption characteristics of these resins under these conditions are shown in Table 7.

**Figure 13:**
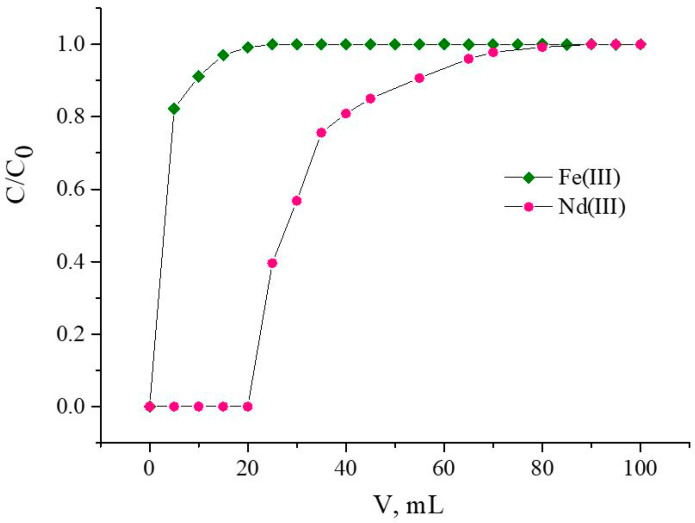
Recovery of Fe(III) and Nd(III) from NH_4_NO_3_ of 1.0 mol/L with resin impregnated by TODGA (SIR 6). The SIR content is 600 ± 3 mg; the concentration of Nd(III) in feed solution is 260.5 ± 0.3 mg/L; the flow rate is 1.0 mL/L.

Additionally, to estimate the separation efficiency of Nd(III) and Fe(III) on a resin impregnated with TODGA (SIR 6), we studied the recovery of these metals from NH_4_NO_3_ with a concentration of 1.0 mol/l (Figure 13).

Under these conditions, the values of Kd for Fe(III) and Nd(III) are 12 ± 4 and 114 ± 7, respectively. Thus, the value of the separation factor of Nd(III)/Fe(III) for SIR 6 is 9 ± 3. At the same time, the value of the separation factor of Nd(III)/Fe(III) for SIR 5 in NH_4_NO_3_ of 1.0 mol/L is 28 ± 2. Therefore, in these conditions, with SIR 5, the separation efficiency of Nd(III)/Fe(III) exceeds SIR 6 almost three times.

### 3.7. Separation of Fe(III) and Nd(III)

**Figure 14:**
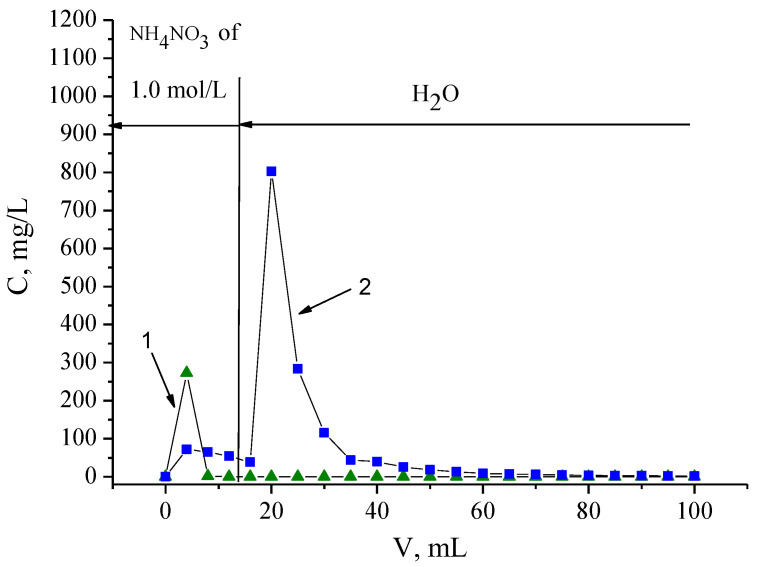
Separation of Fe(III) (1) and Nd(III) (2) using resin SIR 5. The SIR content is 600 ± 3 mg; the concentration of Nd(III) in feed solution is 260.5 ± 0.3 mg/L; the flow rate is 1.0 mL/L.

Based on data presented in Figure 10, an effective approach to separate Fe(III) and Nd(III) is proposed, which includes the preliminary removal of Fe(III) with a washing column packed with resin SIR 5 using an NH_4_NO_3_ solution of 1.0 mol/L, followed by the stripping of Nd(III) with distilled water. The chromatogram obtained under these conditions is shown in Figure 14.

Fe(III) is completely stripped by passing through the column of 8.0 mL of NH_4_NO_3_ with a concentration of 1.0 mol/L, whereas the desorbate obtained contains a small amount of Nd(III) (0.547 mg). The main amount of Nd(III) is stripped by distilled water, and this desorbate is practically important. In our experiment, 6.1 mg of Nd(III) of purity > 99.5%, which is 98.5% of the total amount of loaded Nd(III), was obtained. From the obtained product solution, Nd(III) may be precipitated by (NH_4_)_2_CO_3_ or (NH_4_)_2_C_2_O_4_ [56]. Fe(III), as well as other metals of NdFeB magnets, can be precipitated from ammonium desorbate by adding Na_2_CO_3_. Thus, the production of solid precipitations at both stages of separation allows waste from the separation process to be reduced, which makes it environmentally friendly. Additionally, this approach to the separation of Fe(III) and Nd(III) does not use inorganic acids, which prevents the corrosion of equipment.

### 3.8. Recovery of REE from Nitrate-Based Leachate of NdFeB Magnets

The recovery of Pr(III), Nd(III), and Dy(III) was carried out by using SIR 5 from the leachate of NdFeB, which was preliminarily neutralized using a solution of NH_4_OH to pH ~6.5–7. The results of this experiment are presented in Table 8.

According to Table 8, REEs were almost completely separated from B(III) and all transition metals, which were contained in the leachate of the NdFeB magnet. As result, REE concentrate containing Pr(III), Nd(III), and Dy(III) was obtained. During this process, it was possible to obtain 11.69 mg of REEs with purity of 99.6%, which was 81.6% of the REE mass loaded in the column. Thus, the developed resin demonstrated high efficiency of separation between Fe(III) and Nd(III) in nitrate solutions and is potentially suitable for the recovery of REEs from the leachate of NdFeB magnets.

## 4. Conclusions

A number of new resins impregnated by neutral organophosphorus extractants with different chemical structures were obtained and used for the recovery of rare earth elements from the nitrate-based leachate of NdFeB magnets. The recovery patterns of Fe(III) and Nd(III) from nitric acid solutions using these resins were studied. It was found that the best separation between Fe(III) and Nd(III) is provided by resin impregnated with N,N-dioctyl(diphenylphosphoryl)achetamide (SIR 5). Optimal conditions of separation Nd(III) from Fe(III) using this resin were defined, and an effective method of separation of these metals was suggested. It was established that the value of Nd(III) capacity for developed resin exceeds almost twice the value for resin impregnated by TODGA. The potential suitability of the developed resin for the recovery of Pr(III), Nd(III), and Dy(III) from the nitrate-based leachate of NdFeB magnets was demonstrated. The main advantage of the proposed approach to REE recovery with developed resin is the replacement of acids with electrolyte solutions, which makes it possible to practically eliminate the corrosion of equipment used, reduce the consumption of reagents used for subsequent REE precipitation, and make the process of REE recovery environmentally friendly.

## Figures and Tables

**Table 3:** Compositions of developed resins.

Resin	Compound	Content, wt %
SIR 1	**I**	40.2 ± 0.3
SIR 2	**II**	40.3 ± 0.2
SIR 3	**III**	40.4 ± 0.3
SIR 4	**IV**	40.3 ± 0.2
SIR 5	**V**	40.4 ± 0.3
SIR 6	TODGA	40.3 ± 0.2

**Table 4:** The physical constants of columns packed with resins ^a^.

Resin	Extractant Density, g/mL	Bed Density, (g/cm^3^)	V_s_, mL	V_m_, mL	V_s_/V_m_
SIR 1	1.33 ± 0.03	1.18 ± 0.02	0.24 ± 0.02	1.12 ± 0.02	0.21
SIR 2	1.31 ± 0.02	1.15 ± 0.02	0.23 ± 0.03	1.14 ± 0.02	0.20
SIR 3	1.37 ± 0.04	1.22 ± 0.03	0.23 ± 0.03	1.17 ± 0.03	0.19
SIR 4	1.42 ± 0.04	1.17 ± 0.02	0.25 ± 0.04	1.15 ± 0.02	0.22
SIR 5	1.44 ± 0.05	1.20 ± 0.02	0.24 ± 0.03	1.18 ± 0.03	0.20
SIR 6	1.25 ± 0.03	1.16 ± 0.03	0.22 ± 0.03	1.14 ± 0.02	0.19

^a^ V_s_ is the volume of extractant (mL), which is held by SIR; V_m_ is the volume (mL) of eluent, which is located inside columns packed with SIRs.

**Table 5:** Sorption characteristics of studied resins.

Resin	Kd, mL/g	Capacity, Nd, mg/Resin, 1 g
SIR 1	14 ± 5	1.4 ± 0.2
SIR 2	67 ± 5	6.9 ± 0.3
SIR 3	75 ± 7	7.3 ± 0.3
SIR 4	134 ± 7	12.6 ± 0.5
SIR 5	213 ± 8	18.7 ± 0.4

**Table 6:** The values of Nd(III) distribution coefficients (Kd) and resin capacity in three repeated experiments performed in NH_4_NO_3_ with the concentration of 1.0 mol/L. The SIR content is 600 ± 3 mg; the concentration of Nd(III) in feed solution is 260.5 ± 0.3 mg/L; the flow rate is 1.0 mL/L.

The Experiment Number	Kd, mL/g	Capacity of Resin SIR 5: mg Nd/1.0 g of Resin
1	232	21.2
2	225	21.0
3	226	20.8
Average value	227 ± 10	21.0 ± 0.5

**Table 7:** The values of Nd(III) distribution coefficients and capacity of resins in NH_4_NO_3_ of 1.0 mol/L.

Resin	Kd, mL/g	Capacity, Nd(mg)/1 g Resin
SIR 5	227 ± 10	21.2 ± 0.5
SIR 6	112 ± 7	11.9 ± 0.2

**Table 8:** The values of metal concentrations in product solution.

Element	Concentration, mg/L
Fe	0.21 ± 0.03
Nd	303.1 ± 0.4
B	<detection limit
Co	<detection limit
Pr	47.3 ± 0.2
Dy	4.35 ± 0.02
Al	1.34 ± 0.01
Mn	<detection limit
Cu	0.065 ± 0.01
Ni	<detection limit

## Data Availability

Not applicable.
